# Evaluating the Impact of COVID-19 on Hospital Profit Compensation Activities: A Difference-in-Differences Event Study Analysis in China

**DOI:** 10.3390/healthcare11091303

**Published:** 2023-05-03

**Authors:** Chi Shen, Dan Cao, Qiwei Deng, Sha Lai, Guanping Liu, Liu Yang, Zhonghai Zhu, Zhongliang Zhou

**Affiliations:** 1 School of Public Policy and Administration, Xi’an Jiaotong University, Xi’an 710049, China; 2 School of Public Health, Health Science Center, Xi’an Jiaotong University, Xi’an 710061, China; 3 Center of Health Information of Shaanxi Province, Xi’an 710003, China; 4 The First Affiliated Hospital of Xi’an Jiaotong University, Xi’an 710061, China

**Keywords:** profit compensation activities, COVID-19, hospital, electronic medical records, China

## Abstract

The impact of the 2019 coronavirus disease (COVID-19) pandemic is still being revealed, and little is known about the effect of COVID-19-induced outpatient and inpatient losses on hospital operations in many counties. Hence, we aimed to explore whether hospitals adopted profit compensation activities after the 2020 first-wave outbreak of COVID-19 in China. A total of 2,616,589 hospitalization records from 2018, 2019, and 2020 were extracted from 36 tertiary hospitals in a western province in China; we applied a difference-in-differences event study design to estimate the dynamic effect of COVID-19 on hospitalized patients’ total expenses before and after the last confirmed case. We found that average total expenses for each patient increased by 8.7% to 16.7% in the first 25 weeks after the city reopened and hospital admissions returned to normal. Our findings emphasize that the increase in total inpatient expenses was mainly covered by claiming expenses from health insurance and was largely driven by an increase in the expenses for laboratory tests and medical consumables. Our study documents that there were profit compensation activities in hospitals after the 2020 first-wave outbreak of COVID-19 in China, which was driven by the loss of hospitalization admissions during this wave outbreak.

## 1. Introduction

Since early 2020, the 2019 coronavirus disease (COVID-19) pandemic has raised widespread concern about its socioeconomic impact [1,2,3,4,5,6]. Measures to address COVID-19, such as quarantine, stay-at-home restrictions, or lockdown of cities, have caused significant disruptions to people’s daily life; no one is spared from the shadow of the pandemic. The impact of the COVID-19 pandemic is still being revealed, and numerous studies have reported that the public health measures described above were associated with a marked decrease in health care utilization worldwide, including emergency room visits and hospital admissions for non-COVID-19 patients, such as in Europe [7,8,9,10,11,12,13], Asia [14,15,16], the Americas [17,18,19,20], and Africa [21,22]. However, the COVID-19 pandemic leads to critical hospital response, and the evidence concerning the effect of these responses on hospital operation strategy remains limited.

It is well known that during approximately the past two years, China has responded to the COVID-19 epidemic with persistent and strict measures, including quarantine, stay-behind restrictions, or lockdown of cities, based on the principles of zero COVID-19 or dynamic zero COVID-19 policies, and most countries also adopted these strict measures in the first encounter with COVID-19 in 2020. However, there was a huge difference between China and most other countries in the response to the first outbreak of COVID-19 in early 2020. Most hospital beds were occupied by patients infected with COVID-19 during the first wave in 2020 in many parts of the world, which was different from what happened in most places except Wuhan in China. Benefit from strict lockdown measures during the first outbreak of COVID-19 in 2020, only Wuhan was the epicenter of infection, and there were not many infections in other regions of China [23,24,25,26]. However, during the same period, other regions in China also implemented strict home quarantine policies, and residents had to go through a rigorous approval process to seek medical treatment at hospitals. Additionally, a large number of medical personnel were deployed to conduct nucleic acid testing, leading to a shortage of hospital staff. As a result, outpatient and inpatient volumes in hospitals in areas other than Wuhan showed a significant decrease.

There are serious concerns about how hospitals in China would respond to the increased costs and lost revenue of fighting COVID-19, and whether hospitals will compensate for lost revenue by inducing more diagnostic tests and medical consumables to achieve better financial sustainability. Therefore, this study aims to explore empirically whether hospitals adopted profit compensation activities after the 2020 first-wave outbreak of COVID-19 in China. The profit compensation activities we propose above refer to a series of actions taken by hospitals to increase medical revenues to address financial sustainability goals when they experience declining occupancy and revenue [27].

The importance of paying attention to changes in hospital operation strategy is that it is directly associated with the quality of care and the economic burden on patients, understanding these changes will facilitate the development of policies to prevent a decline in the quality of care and an increase in the economic burden of disease. Along with the decline in outpatient visits and hospital admissions of hospitals, there are losses of revenue that can affect hospitals’ operational strategy, especially for countries like China that have a mix of public and private hospitals in their healthcare delivery systems.

In this study, we applied a difference-in-differences (DID) event study analysis using hospitalization electronic medical records (EMR) from 36 tertiary general hospitals (highest-grade medical institution in China) in a western province of China. We found that average total expenses for each patient increased by 8.7% to 16.7% in the first 25 weeks after the city reopened and hospital admissions returned to normal. Our findings contribute to the so far rich literature by exploring the impact of the COVID-19 pandemic on the health sector.

The rest of this paper is organized as follows. Section 2 introduces the background of China’s healthcare delivery system; Section 3 describes data source, study design, and causal inference strategy; Section 4 and Section 5 present results and discuss the possible interpretation of profit compensation activities in hospitals; and Section 6 concludes the study.

## 2. Background

### 2.1. Brief Introduction to China’s Healthcare Delivery System

China’s healthcare delivery system is a complex network of public and private providers, governed by policies and regulations aimed at ensuring access to basic health services for its large and diverse population. China has a three-tiered system for healthcare delivery: health organizations and providers operate at county, township, and village levels in rural areas, and at municipal, district, and community levels in urban areas [28]. Primary care services are provided at community health centers and township healthcare centers, secondary care is provided by district and county hospitals, and tertiary care is provided by large specialty and general hospitals in major cities.

Public hospitals are owned and operated by the government, and are organized into tiers according to their level of service. They are typically the largest and most well-funded providers, with strong links to medical education and research institutions. Public hospitals dominate the market for specialized and tertiary care, and employ 64% of practicing physicians, handle 82% of inpatients and 40% of outpatients, and account for about half of China’s total healthcare spending [29].

Private hospitals, on the other hand, are owned and operated by nongovernmental entities or individuals. They are generally smaller and provide more specialized services than public hospitals, such as cosmetic and reproductive medicine. Historically, private hospitals have been less regulated than their public counterparts, but recent reforms have sought to improve their quality and increase their role in the healthcare delivery system. The private sector has experienced a vast expansion in China’s hospital market in the past decades for both healthcare supply capacity and delivered care [30], but the public health sector is the main healthcare provider [28].

### 2.2. Compensation Mechanism of Chinese Public Hospitals and Its Potential Influence by COVID-19

Public hospitals in China have been mainly compensated by service charges, drug sales, and government budget allocations in the past decades. However, since 2012, the compensation mechanism of Chinese public hospitals has undergone a relatively large reform, starting with county-level public hospitals and extending to municipal-level public hospitals in 2016. This round reform focused on four interrelated areas: removing the drug mark-up, increased budget allocation, adjustments of fee schedules, and reforming payment methods [29], as a consequence that public hospitals revenue from service charges and government budget allocations only. Moreover, removing the up to 15% markup for drug sales from public hospitals in China brought about a reduction in drug expenditures expected by the policymakers [31,32,33,34,35,36], however, an increase in expenditures for diagnostic tests or medical consumables and hospitalization was observed from different aspects [31,32,33,35], which was not expected by the policymakers, and government subsidies to public hospitals increased slightly by 2–5% in the 2 to 6 years after the policy was implemented [36]. All this evidence suggests that China’s public hospitals rely heavily on medical service charges to sustain their operations and development.

**Figure 1:**
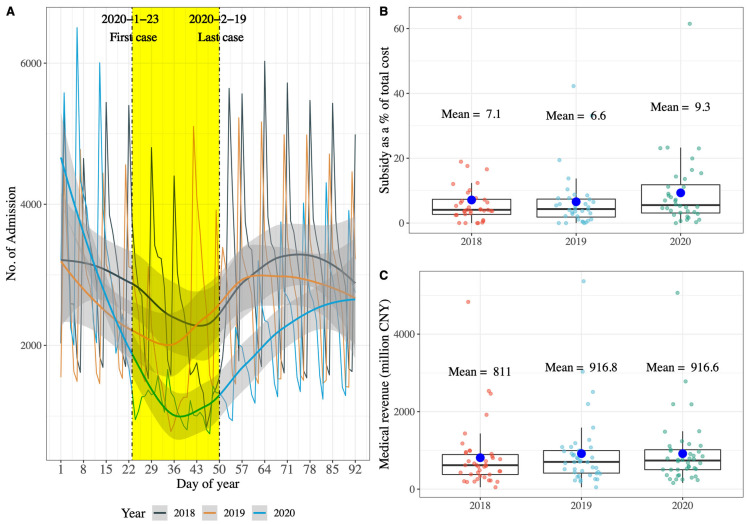
Comparing of number of hospitalization admissions, subsidy share of total cost, and medical revenue for 36 tertiary hospitals from 2018 to 2020. CNY: Chinese yuan; The solid curves in plot (**A**) are the smoothed curves fitted with the locally weighted regression, and the gray shaded area is the confidence interval of the fitted curve; Data source of plot (**B**) and plot (**C**): 36 public tertiary hospitals and 42 tertiary hospitals (including 6 private and 36 public hospitals) from Annual Report on Health Statistics of X Province, respectively.

As mentioned, hospitals in many countries experienced an admission loss during the first encounter with COVID-19 in 2020; we also observed a significant admission loss of tertiary hospitals in China using our data, in line with other studies (Figure 1A) [14,16]. In addition, we found that public hospitals of these tertiary hospitals in China received more subsidies for the loss of service charges caused by the admission loss; government subsidies as a percentage of total costs increased from 6.6% in 2019 to 9.3% in 2020 (Figure 1B), however, it is complicated because medical revenue of tertiary hospitals did not decrease between 2019 and 2020 (Figure 1C). Therefore, we hypothesize that tertiary hospitals in China have profit compensation motive and activities when observing that the admission count returned to normal after strict measures were relaxed.

## 3. Methods

### 3.1. Data

We originally collected hospitalization EMR at patient level from 42 local tertiary general hospitals in a western province of China from 1 January to 31 December in 2018, 2019, and 2020. The 42 local tertiary general hospitals covered all of the 10 prefectural cities in the western province. Disclosure of the province’s name (hereinafter referred to as X Province) is not permitted due to a confidentiality agreement, but it is available from the corresponding authors on reasonable request.

A total of 3,820,359 (1,294,897 in 2018, 1,379,538 in 2019, 1,145,942 in 2020) hospitalization records were initially submitted by the 42 hospitals; we excluded records without any detailed demographic, diagnostic, and expense information and any outliers in these detailed indicators, along with records of patients admitted between 23 January and 19 February (we explain the reason in the next section). We examined the differences in demographics and hospitalizations between the original and cleaned datasets and showed no differences. (Please see Table S1 in Supplementary Materials). Furthermore, considering that the hospitals in China usually reject patients with medical insurances at the end of the year when they reach the medical insurance budget cap [37], which has a significant impact on the average expenses for hospitalized patients, we excluded records of patients admitted in December. Finally, a total of 2,616,589 (889,102 in 2018, 884,408 in 2019, 843,079 in 2020) EMR from 36 local tertiary general hospitals, including 5 private and 31 public hospitals, were included in final analysis. The flowchart of sample selection is shown in Supplementary Figure S1.

Patient data included information on demographics (e.g., age, sex, etc.), disease situation (e.g., primary diagnosis, ICD-10 code, etc.), treatment (e.g., admission date, discharge date, length of admission, etc.), and all kinds of treatment and examination expenses (e.g., total expenses, out-of-pocket, laboratory examination and medical consumables expense, etc.). The basic information of patients included in our study is described in Table 1.

### 3.2. Intervention Identification

Based on the publications [38,39,40] and official statistic website (https://2019ncov.chinacdc.cn/2019-nCoV/, accessed on 15 September 2022), we found the first and the last confirmed case with COVID-19 in the first wave of the 2020 outbreak in X Province were reported on 23 January and 19 February 2020, respectively. In response, local governments in X Province took a series of measures to mitigate the spread of the virus as soon as the first cases were confirmed, including stay-at-home restrictions or lockdowns of cities. However, we attempted but failed to extract detail about the stay-at-home restrictions or lockdowns interventions in the 10 prefectural cities and the exact timing of their implementation from official sources. Nevertheless, we observed the impact of the intervention and its duration in a different way: daily hospitalization admissions of hospitals. As shown in Figure 1A, from 23 January to 19 February 2020, the daily counts of hospitalization admissions at the 36 tertiary general hospitals decreased significantly compared to the same period in 2018 and 2019. In the context of the Chinese’s hospitalization in tertiary hospitals increasing yearly, the sudden decrease in the number of daily hospitalization admissions cannot be reasonably explained by normal policy, unless it is COVID-19.

After 19 February 2020, X Province experienced a long period of zero COVID-19 and these interventions have been relaxed since then. Therefore, we considered the period from 23 January to 19 February 2020 as the treatment phase, the days after 19 February 2020 were the effect period (treatment period) by the interventions of stay-at-home restrictions or lockdowns against COVID-19, the days before 23 January 2020 were the nonimpact period (nontreatment period). We are interested in whether the hospitals had a profit compensation motive or activities when the city reopened and the hospitals returned to normal. In other words, the difference between treatment period and nontreatment period reflects the change in the hospital’s operation strategy.

### 3.3. Causal Inference Strategy

A DID event study design was applied in our study, which is a popular and useful tool in evaluating treatment effects of the pre- and post-treatment periods and widely used in empirical studies in economics. DID event study design is a derivative form of the standard DID and is very powerful in estimating treatment effects for a staggered situation where units are treated at different times. Although in our study the intervention of stay-at-home restrictions or lockdowns against COVID-19 were performed at the same time rather than staggered, DID event study design was also applicable and provided a clear estimate of the treatment effect.

**Figure 2:**
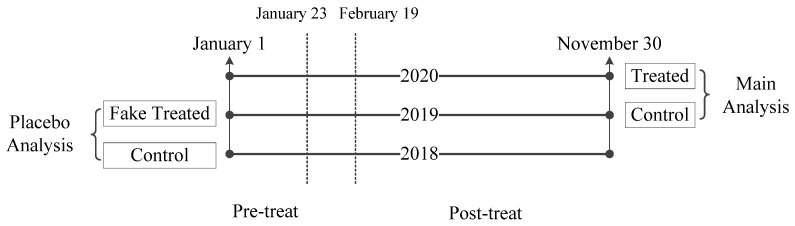
Framework of casual inference strategy in the study.

According to DID event study design, the framework of causal inference strategy is presented in Figure 2. Since all provinces in China were affected by COVID-19 to a greater or lesser extent, it was not possible to find hospitals from other provinces or within X province that were similar to our 36 sample hospitals but not affected by COVID-19. Therefore, we selected the hospitalization electronic medical records from the same 36 sample hospitals in 2019 as the control group for 2020 in main analysis; although not perfect, it has been proved that records from an adjacent year may be an appropriate control group in some studies using a DID event study design that is based on electronic medical records [41] and other types of longitudinal survey data [42,43].

Considering that the estimated treatment effect in 2020 may be caused by potentially unobserved treatments (e.g., natural increase in costs accompanying consumer price index) instead of COVID-19, we performed a placebo analysis with 2019 for the treatment group and 2018 for the control group to estimate a placebo treatment effect for 2019 (faked treated). The insignificant treatment effect of the placebo analysis would address concerns about potential unobserved bias in the context of the assumption that no exogenous policies affecting hospitalization costs were introduced during the three years from 2018 to 2020.

In addition, as previously mentioned, we dropped records of patients admitted between 23 January and 19 February because the 36 sample hospitals were in nonfunctional status during this period, which may have resulted in significantly different hospitalization conditions and hospitalization costs during this period because most were set up as designated hospitals for COVID-19-infected patients in their municipalities and some physicians were involved in outbreak prevention and control.

The equation applied to our DID event study is:

(1) $$log\left(Expense_{it}\right)=\alpha +\sum_{k=T_{0}}^{-2}\beta_{k}\cdot Treat_{ik}+\sum_{k=0}^{T_{1}}\beta_{k}\cdot Treat_{ik}+X_{it}\cdot \tau +\gamma_{t}+\delta_{h}+\varepsilon_{it}$$

where:

- $log\left(Expense_{it}\right)$ denotes the logarithmic form of total expenses, out-of-pocket expenses, and claiming expenses from health insurance for each patient *i* admitted at week *t*.
- $T_{0}$ and $T_{1}$ are the lowest and highest numbers of week of leads and lags to consider for the period of week 4 to week 8 of the year (23 January to 19 February). The value of *k* is −1, −2, $T_{0}$ when the week of the year is 3, 2, 1. The value of *k* is 0, 1, ⋯, $T_{1}$ when the week of the year is 8, 9, ⋯, 48 (30 November).
- $Treat_{ik}$ is a dummy variable equaling 1 if the observation is from 2020, otherwise it is 0.
- $\gamma_{t}$ and $\delta_{h}$ are week and hospital fixed effects.
- $X_{it}$ represent a series of control variables that would have significant confounding effects on inpatient expense, including age, age square, sex, type of health insurance, admission situation, and first ICD-10 code for primary diagnosis.
- Estimation is generally performed with standard errors clustered at the hospital level.
- The −1 week lag is used as the dropped reference to avoid perfect multicollinearity.

Commonly, two-way fixed effects (TWFE) is the preferred choice in DID design or event study design; in recently years, however, some studies [44,45,46,47] argued that TWFE cannot always provide unbiased estimation under staggered treatment or policy rollout. Although there is no staggered intervention in our study, we applied the method proposed by Sun and Abraham (2020) [44] to estimate the $\beta_{k}$ in Equation (1); the $\beta_{k}$ coefficients test the impacts of hospitalization admission losses on the hospitals’ profit compensation activities in the weeks before and after the COVID-19 outbreak.

## 4. Results

### 4.1. Descriptive Analysis

**Figure 3:**
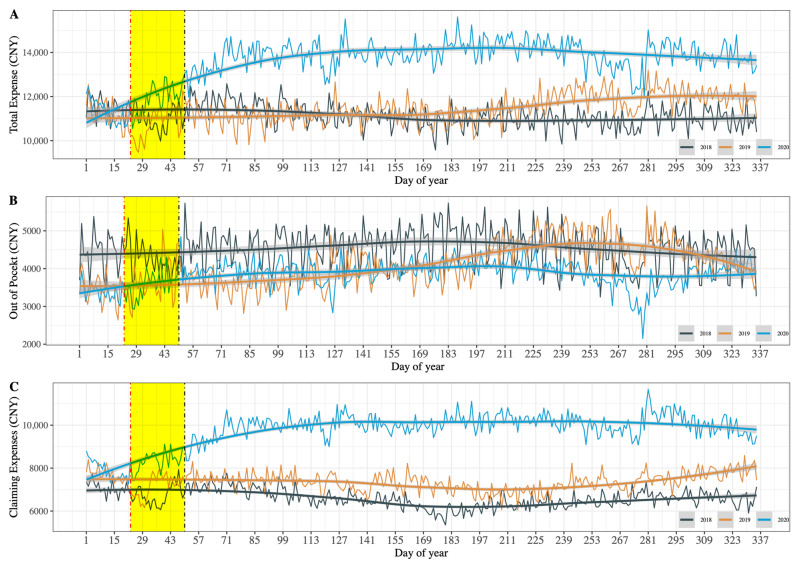
Comparing the trend of total expenses, out-of-pocket, and claiming expenses for hospitalized patients in 36 tertiary hospitals from 2018 to 2020, aggregate mean value at admitted date. CNY: Chinese yuan; The solid curves are the smoothed curves fitted with the locally weighted regression, and the gray shaded area is the confidence interval of the fitted curve.

We first focused on the trends in total expenses, out-of-pocket expenses, and claiming expenses for each day of admissions from 23 January to 19 February in 2018, 2019, and 2020; the average of patients’ expenses for each day’s admissions were calculated. As shown in Figure 3A, a clear gap was observed between 2020 and 2019 after 19 February when the last case with COVID-19 in the first wave of the 2020 outbreak was confirmed; however, no such gap existed between 2019 and 2018 during the same period or even for most of the year. Furthermore, we divided total expenses for patients into out-of-pocket and claiming expenses to explore which one contributed to the gap in total expenses between 2020 and 2019. Results shown in Figure 3B,C indicate that increase in total expenses after the stay-at-home restrictions or lockdowns interventions were relaxed may be primarily attributable to the increase in claiming expenses rather than out-of-pocket. We can learn from the descriptive analysis results that hospitals in China did take profit compensation activities after nearly a month of lost revenue during the first wave of the COVID-19 outbreak, as we hypothesized.

### 4.2. DID Event Study Analysis

**Figure 4:**
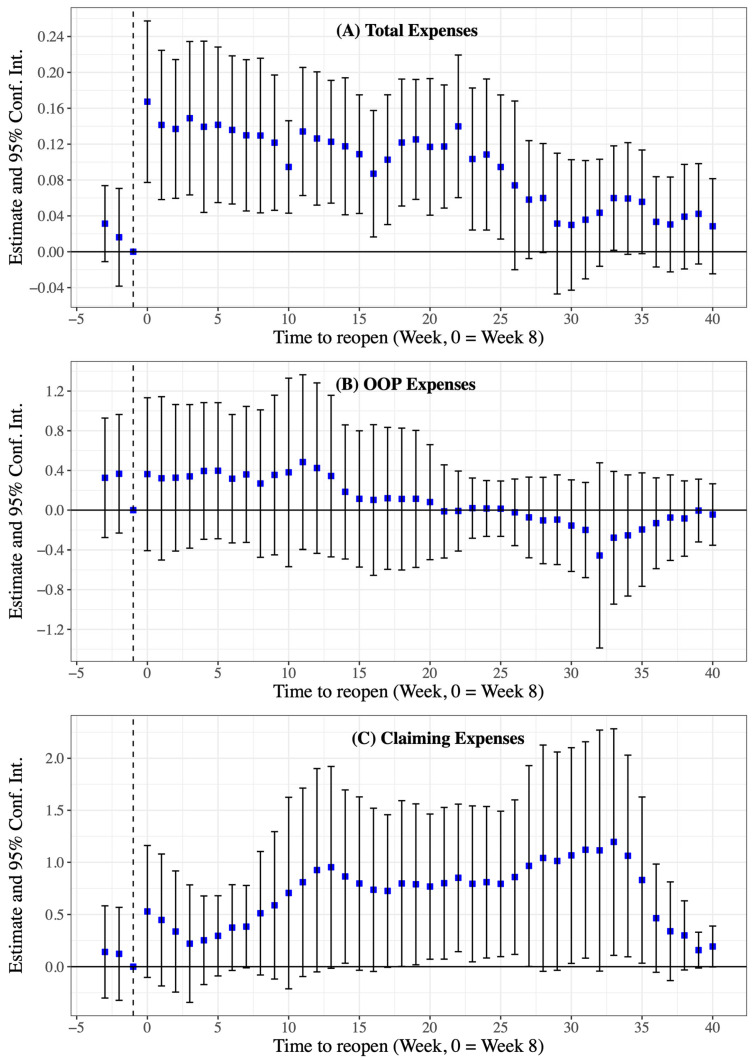
Dynamic estimates and 95% Conf. Int. for total expenses, out-of-pocket (OOP) expenses, and claiming expenses for hospitalized patients in 36 tertiary hospitals between 2019 and 2020 (main analysis). Blue dots are the estimates, black solid lines are 95% Conf. Int. for point estimates, and expenses are log-transformed.

We were interested in how much profits compensation the hospitals received from the hospitalization patients’ costs and claiming expenses. Concerning the event study estimation, there are three more features that we can observe from the descriptive results. First, the increase in total inpatient expenses showed an overall downward trend, characterized by a two-phase approach before and after 25 weeks following the city reopening and hospital admissions returning to normal. The estimated profit compensation effects in the event study were 8.7% (95% Conf. Int. 1.7% to 15.8%) to 16.7% (95% Conf. Int. 7.7% to 25.7%) in the first 25 weeks and 2.8% (95% Conf. Int. 2.5% to 8.2%) to 7.4% (95% Conf. Int. 2.0% to 16.9%) after the first 25 weeks (Figure 4A). Second, we confirmed that the profit compensation activities did not take from the patients’ OOP expenses (Figure 4B). Third, the estimated profit compensation effects presented a complex tread, but the impact of estimated profit compensation on claims expenses was greater than the impact on total expenses (Figure 4C). The regression results linked to Figure 4 can be found in Supplementary Table S2.

**Figure 5:**
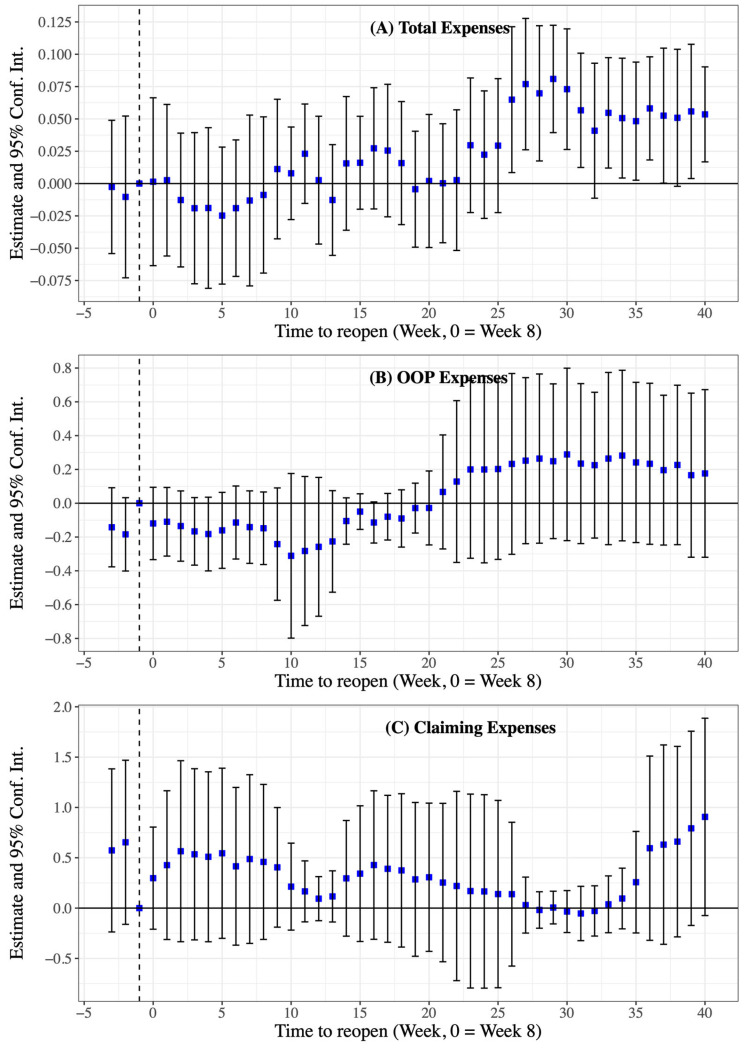
Dynamic estimates and 95% Conf. Int. for total expenses, out-of-pocket (OOP) expenses, and claiming expenses for hospitalized patients in 36 tertiary hospitals between 2018 and 2019 (placebo analysis). Blue dots are the estimates, black solid lines are 95% Conf. Int. for point estimates, and expenses are log-transformed.

Moreover, results in the placebo test show that there were no significant changes in hospitalization patients’ expenses between 2018 and 2019 around the period that the city reopened and hospital admissions returned to normal (Figure 5), which increases the confidence in attributing the unusual increase on patients’ expenses after 19 February 2020 to the hospitalization admission losses during the fight against COVID-19.

### 4.3. Heterogeneous and Sensitivity Analysis

**Figure 6:**
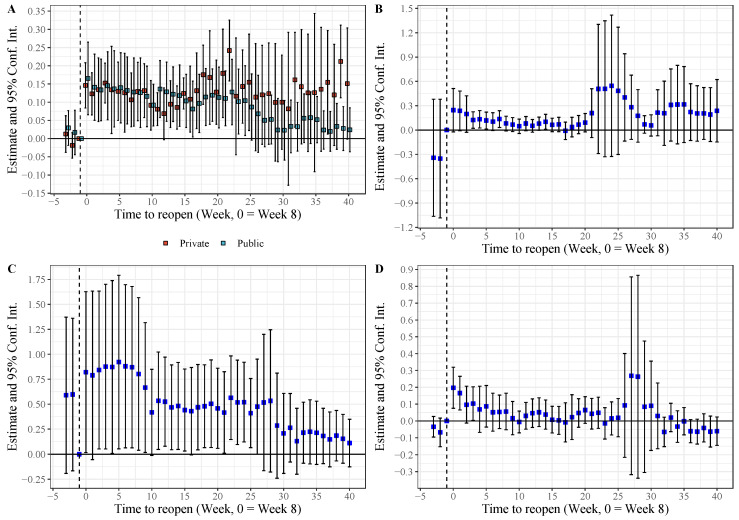
Heterogeneity analysis: (**A**) dynamic estimates and 95% Conf. Int. for total expenses for hospitalized patients in 5 private and 31 public tertiary hospitals between 2019 and 2020; (**B**) dynamic estimates and 95% Conf. Int. for laboratory test expenses for hospitalized patients in 36 tertiary hospitals between 2019 and 2020; (**C**) dynamic estimates and 95% Conf. Int. for medical consumables expenses for hospitalized patients in 36 tertiary hospitals between 2019 and 2020; (**D**) dynamic estimates and 95% Conf. Int. for drug expenses for hospitalized patients in 36 tertiary hospitals between 2019 and 2020. Blue dots are the estimates, black solid lines are the 95% Conf. Int. for point estimates, and expenses are log-transformed.

We were also interested in two other questions: whether private hospitals have stronger profit compensation activities than public hospitals, and how public hospitals can increase patient expenses under strict supervision of medical expenses by health administration and medical insurance agencies; we conducted additional analysis to address these questions. As shown in Figure 6A, total inpatient expenses generally increased more in the 5 private hospitals than in the 31 public hospitals, which may be due to the lack of government subsidies in the private hospitals, forcing them to make up more of their lost profits from medical revenues.

To determine what kinds of expenses contributed to the increase in total expenses for the public hospitals, we examined the expenses for laboratory tests, drugs, and medical consumables. As shown in Figure 6, expenses for laboratory tests and medical consumables showed continued significant growth in line with the total expenses. In addition, we found that a higher proportion of patients had surgery after cities reopened in 2020 than in 2018 and 2019, and that a high proportion of surgeries may also boost hospitalization costs (see Supplementary Figure S2).

## 5. Discussion

This study documents that there were profit compensation activities in hospitals after the 2020 first-wave outbreak of COVID-19 in China. The profit compensation motive was driven by the loss of hospitalization admissions during this wave outbreak, specifically, the profit compensation activities were manifested as an increase in total inpatient expenses of 8.7% to 16.7%, which was largely driven by an increase in the expenses for laboratory tests and medical consumables. To a certain extent, the good news for patients is that the hospitals’ profit compensation activities have not aggravated the OOP expenses for patients. These findings indicate that the loss of revenue accompanying the loss of hospitalization admissions worried hospital administrators, which led them to pursue more revenue from medical charges, even challenging the regulations from medical insurance regulators.

We suspect that the profit compensation motive of China’s public hospitals is mainly due to the compensation strategy that relies heavily on medical service fees, and also due to the fear of a shock to normal hospital operations from the next wave of COVID-19 outbreaks in an uncertain future. Data from National Health Commission of China (NHCC) show that government subsidies shared between 8% and 10% of total public hospital costs during 2010 and 2019 [48] with the remainder being borne by hospitals through charges from medical services and efficiency gain from improved management [29]. The facts force hospital administrators to watch over the hospital revenue situation; compensatory measures are taken to increase revenue once revenue falls below expectations, especially when facing the widespread impacts from the novel COVID-19 outbreak.

Our results show, however, that the percentage of government subsidies of public hospital costs increased from 6.6% in 2019 to 9.3% in 2020, which means additional government subsidies were paid to public hospitals, so why did hospitals still charge more for medical services? We infer that hospitals did not know in advance that the government would provide additional subsidies; therefore, concerns about the loss of revenue were not eliminated at that time. NHCC and China’s Ministry of Finance jointly issued a document on 25 January 2020 [49], stating that the treatment costs of patients infected with COVID-19 were covered by both health insurance and finance, but there is no mention that there would be additional subsidies for designated hospitals.

The increase in total inpatient expenses may be attributed to other patient-side mechanisms instead of profit compensation activities, as we proposed; however, we argue that the impact of these mechanisms may be weaker than the profit compensation activities.

First, patients admitted to the hospital in the first weeks after the city reopened may have had more severe cases, which were caused by nearly a month of hospitalization admission restrictions due to stay-at-home restrictions or lockdowns [50], as seen in the higher rate of admissions from emergency room visits in 2020 (Figure S3 in Supplementary Materials), and the more severe cases increased the medical expenses. Specifically, the impact of stay-at-home restrictions or lockdowns on causing more severe cases may result from the fact that many patients with cardiovascular disease (CVD) could not be admitted to the hospital normally. Evidence from several countries suggests that there was a significant decrease in admissions or visits to health care facilities for CVD in 2020 compared to previous years [51,52,53], and evidence from China also shows that the emergency attendance rates for stroke and acute myocardial infarction were cut in half at the beginning of the lockdown period [54]. However, these effect of these severe cases cannot lead to a long-term increase in medical expenses, and the proportion of severe cases among hospitalized patients may decrease to normal after a few weeks when hospital capacity was restored; therefore, the increase in medical expenses up to 25 weeks that we observed in our findings cannot be fully explained by the effect of the severe cases. On the other hand, the higher rate of admissions from emergency room visits may be due not only to the more severe disease conditions of the patients, but also to the government’s requirement that hospitals routinely close outpatient services and open only the emergency department in order to prevent the spread of COVID-19.

Second, the high rate of avoidable hospitalizations associated with low-quality primary care is a challenge that China has struggled to address [55,56]; additional hospitalization admissions requirement for patients during the fight against COVID-19, such as the requirement for negative nucleic acid tests within 24 or 48 h and other entrance limitations for patients and their companions, may filter out avoidable hospitalizations, thereby reducing the proportion of hospitalized patients with minor illnesses and thus increasing the average expenses for hospitalization. However, the effect of these avoidable hospitalizations should lead to a decrease in hospitalization admissions, but which was shown in our findings to have increased.

Moreover, not surprisingly, we found increases in the expenses for laboratory tests and medical consumables and the proportion of patients undergoing surgery were the main reasons for the increase in hospitalization expenses, which is consistent with the findings of other studies [32,33]. This implies that the path of increasing health care costs through expenditures for diagnostic tests and medical consumables has been uninterrupted since public hospital reform in China during 2016. Certainly, the increase in the proportion of patients undergoing surgery was not necessarily induced by the hospital’s profit compensation activities, but may also be due to the promotion of day surgery in many hospitals in China during the COVID-19 pandemic; the convenience of day surgery increases the volume of surgeries [57].

Furthermore, it is somewhat unusual to observe an increase in total inpatient expenses but not in OOP expenses. We think this may be caused by an increase in inpatient admissions with low patient cost-sharing; however, we were unable to provide evidence of this in this study, further studies are needed to explore it. Additionally, we suggest such a result is also reasonable because medical insurance reimbursement covers most of the hospitalization costs in China, and medical insurance in China does not use a fixed percentage of reimbursement, but rather a dynamic variation in reimbursement rates with the use of different treatments and medications, averaging 64.9% in tertiary hospitals [58]. As a result of the COVID-19 pandemic, China’s medical insurance has covered most treatment costs for COVID-19 patients, leading to increased medical insurance spending and consequently increased regulation of hospital use for medical insurance [59]. Physicians may be more compliant with medical insurance reimbursement rules when treating patients. Therefore, an increase in total inpatient expenses does not necessarily result in an increase in OOP expenses.

Our findings suggest that governments should take more responsibility for declining hospital admissions and lost revenue due to public policy, such as city lockdowns to address COVID-19, because this loss of admissions and reduction in revenue was not due to poor hospital operation. Otherwise, hospital revenue compensation activities will eventually shift this loss to patients and society. The government’s responsibility should specifically be giving hospitals adequate subsidies and informing them of this information in a timely manner, together with creating a good expectation for hospitals.

Regarding limitations, total health expenditure was growing at an average annual rate of 12.2% in China [60]; this growth may be included in our estimates of hospitalization expenditures in tertiary hospitals, although we applied a DID event study design. Nevertheless, the estimates of increased hospitalization expenditures in our study can still reflect the profit compensation activities in tertiary hospitals, because the estimates in our placebo analysis demonstrate that the 12.2% increase in total healthcare spending did not result in a significant difference between 2018 and 2019, and then, we believe, between 2020 and 2019 as well. Moreover, the hospital’s electronic medical records did not include exact claiming expenses for each patient with medical insurance; the claiming expenses used in our analysis were derived from the difference between total expenses and OOP expenses for each patient, which may not fully reflect the true claim expenses due to China’s complex claims rules. We are confident, however, that this does not have a serious effect on the veracity of our conclusions. Furthermore, our study does not provide strong empirical evidence to exclude increases in hospitalization expenses related to patient characteristics, such as more severe disease conditions, and further studies are needed to validate this.

## 6. Conclusions

Our study has shown that tertiary hospitals in China experienced nearly one month of hospitalization admission losses during the 2020 first-wave outbreak of COVID-19, and these hospitals adopted profit compensation activities that increased the total inpatient expenses from 8.7% to 16.7% up to 25 weeks after the 2020 first-wave outbreak. Our findings emphasize that the increase in total inpatient expenses was mainly covered by claiming expenses from health insurance and was largely driven by an increase in the expenses for laboratory tests and medical consumables. Our study emphasizes that government should pay more attention to the long-term impacts of COVID-19 on hospitals, and take more responsibility for hospital losses. This could be accomplished by establishing a series of public policies aimed at mitigating the spread of the COVID-19, which would include adequate subsidies and informing hospitals of this information in a timely manner, in order to avoid losses being passed on to patients and society.

## Figures and Tables

**Table 1:** Demographic and expense characteristics of hospitalized patients in the study.

Indicators	2018 (*n* = 889,102)	2019 (*n* = 884,408)	2020 (*n* = 843,079)	Overall (*n* = 2,616,589)
Age (year)				
Mean (SD)	50.1 (22.4)	50.1 (22.5)	51.8 (21.2)	50.7 (22.1)
Median [Min, Max]	54.0 [1.00, 119]	54.0 [1.00, 110]	56.0 [1.00, 107]	55.0 [1.00, 119]
Sex				
Male	421,330 (47.4%)	416,751 (47.1%)	414,921 (49.2%)	1,253,002 (47.9%)
Female	467,772 (52.6%)	467,657 (52.9%)	428,158 (50.8%)	1,363,587 (52.1%)
Insurance Payment				
UEBMI	260,256 (29.3%)	266,725 (30.2%)	266,296 (31.6%)	793,277 (30.3%)
URBMI	115,294 (13.0%)	125,926 (14.2%)	227,737 (27.0%)	468,957 (17.9%)
NRCMSI	304,335 (34.2%)	276,585 (31.3%)	129,458 (15.4%)	710,378 (27.1%)
Poverty relief	5004 (0.6%)	6468 (0.7%)	10,365 (1.2%)	21,837 (0.8%)
Commercial medical insurance	902 (0.1%)	751 (0.1%)	670 (0.1%)	2323 (0.1%)
Publicly funded medical care	2869 (0.3%)	2001 (0.2%)	1855 (0.2%)	6725 (0.3%)
No medical insurance	135,812 (15.3%)	165,384 (18.7%)	127,847 (15.2%)	429,043 (16.4%)
Other social medical insurance	8023 (0.9%)	9693 (1.1%)	6949 (0.8%)	24,665 (0.9%)
Other insurance	56,607 (6.4%)	30,875 (3.5%)	71,902 (8.5%)	159,384 (6.1%)
Type of Admission				
Emergency	228,982 (25.8%)	226,141 (25.6%)	270,103 (32.0%)	725,226 (27.7%)
Outpatient	622,129 (70.0%)	614,395 (69.5%)	557,553 (66.1%)	1,794,077 (68.6%)
Referrals from other institutions	1018 (0.1%)	1355 (0.2%)	1785 (0.2%)	4158 (0.2%)
Other	36,973 (4.2%)	42,517 (4.8%)	13,638 (1.6%)	93,128 (3.6%)
Surgical				
No	515,599 (58.0%)	479,911 (54.3%)	408,859 (48.5%)	1,404,369 (53.7%)
Yes	373,503 (42.0%)	404,497 (45.7%)	434,220 (51.5%)	1,212,220 (46.3%)
Length of Admission				
Mean (SD)	9.67 (6.97)	9.32 (6.91)	9.58 (6.88)	9.52 (6.92)
Median [Min, Max]	8.00 [1.00, 90.0]	8.00 [1.00, 90.0]	8.00 [1.00, 90.0]	8.00 [1.00, 90.0]
Total Inpatient Expenses (CNY)				
Mean (SD)	11,300 (12,800)	11,600 (13,500)	13,700 (16,400)	12,200 (14,300)
Median [Min, Max]	7070 [825, 90,100]	7200 [768, 94,700]	7860 [782, 108,000]	7350 [768, 108,000]
Out-of-Pocket (CNY)				
Mean (SD)	4690 (10,400)	4200 (9100)	3870 (9200)	4260 (9590)
Median [Min, Max]	0 [0, 90,100]	0 [0, 94,700]	0 [0, 108,000]	0 [0, 108,000]
Claim Expenses (CNY)				
Mean (SD)	6590 (10,300)	7410 (11,300)	9840 (14,200)	7920 (12,100)
Median [Min, Max]	3800 [0, 90,100]	4280 [0, 94,700]	5460 [0, 108,000]	4520 [0, 108,000]
Laboratory Test Expenses (CNY)				
Mean (SD)	1050 (1090)	1180 (1150)	1290 (1280)	1170 (1180)
Median [Min, Max]	830 [0, 35,000]	943 [0, 47,600]	1010 [0, 37,200]	923 [0, 47,600]
Imaging Test Expenses (CNY)				
Mean (SD)	732 (1330)	768 (1360)	909 (1570)	801 (1420)
Median [Min, Max]	331 [0, 58,300]	330 [0, 58,600]	435 [0, 70,700]	369 [0, 70,700]
Drug Expenses (CNY)				
Mean (SD)	2850 (4140)	2740 (3960)	3120 (4520)	2900 (4210)
Median [Min, Max]	1580 [0, 73,900]	1570 [0, 86,600]	1750 [0, 86,100]	1630 [0, 86,600]
Medical Consumables Expenses (CNY)				
Mean (SD)	1440 (5420)	2220 (7210)	3310 (9350)	2310 (7500)
Median [Min, Max]	0 [0, 85,300]	85.1 [0, 86,500]	163 [0, 100,000]	79.9 [0, 100,000]

Note: CNY: Chinese yuan, UEBMI: Urban Employment-based Basic Medical Insurance, URBMI: Urban Resident-based Basic Medical Insurance, NRCMSI: New Rural Cooperative Medical Scheme Insurance.

## Data Availability

The data used in this study originate from the Health Information Center of X Province. There are restrictions on the availability of this data and therefore it is not publicly available. All analyses were performed using the statistical software R version 4.1.3. Data and code to reproduce all figures and statistical analyses are available from the corresponding authors on reasonable request.

## Supplementary Materials

The following supporting information can be downloaded at: https://www.mdpi.com/article/10.3390/healthcare11091303/s1, Figure S1: Flowchart of the study sample; Figure S2: Percentage of patients surgery among 2018, 2019 and 2020; Figure S3: Percentage of admission from emergency room visit among 2018, 2019 and 2020; Table S1: Comparison of demographic and hospitalization between raw and cleaned dataset; Table S2: Regression results of difference-in-difference event study based on data of 2019 and 2020.
