# Supplementary material for: Evaluating the Impact of COVID-19 on Hospital Profit Compensation Activities: A Difference-in-Differences Event Study Analysis in China
Source: Healthcare (Basel). 2023 May 3;11(9):1303. doi: 10.3390/healthcare11091303 (PMC10178599; doi:10.3390/healthcare11091303)
Supplement: Supplementary file 1 [file healthcare-11-01303-s001.zip › healthcare-2309345-supplementary.pdf]

Supplementary

Table S1. Comparison of demographic and hospitalization between raw and cleaned dataset

Characteristic	2020		2019		2018	
	Clean Dataset, N = 947,513	Raw dataset, N = 1,145,924	Clean Dataset, N = 1,021,386	Raw dataset, N = 1,379,538	Clean Dataset, N = 1,016,493	Raw dataset, N = 1,294,897[1]
Age (yr)	55 (36, 68)	55 (36, 67)	54 (33, 67)	54 (33, 67)	54 (33, 67)	54 (33, 67)
Total Hospitalization Expenses (CNY)	7,772 (4,594, 14,388)	7,723 (4,521, 14,272)	7,157 (4,315, 12,359)	7,148 (4,283, 12,267)	7,016 (4,190, 12,195)	7,015 (4,173, 12,200)
Sex						
Male	465,223 (49%)	564,596 (49%)	480,276 (47%)	657,396 (48%)	480,494 (47%)	618,991 (48%)
Female	482,290 (51%)	581,328 (51%)	541,110 (53%)	722,142 (52%)	535,999 (53%)	675,906 (52%)
Admission Way						
Emergency	305,314 (32%)	432,128 (38%)	260,116 (25%)	417,812 (33%)	264,044 (26%)	416,050 (33%)
Outpatient	624,955 (66%)	650,590 (57%)	708,378 (69%)	753,487 (59%)	709,628 (70%)	747,673 (59%)
Referrals from other institutions	2,067 (0.2%)	2,111 (0.2%)	1,526 (0.1%)	1,591 (0.1%)	1,174 (0.1%)	1,248 (<0.1%)
Other	15,177 (1.6%)	61,095 (5.3%)	51,366 (5.0%)	111,340 (8.7%)	41,647 (4.1%)	96,061 (7.6%)
Missing			0	95,308	0	33,865
Insurance Payment						
UEBMI	295,969 (31%)	372,778 (33%)	306,697 (30%)	409,357 (30%)	296,285 (29%)	374,435 (29%)
URBMI	258,618 (27%)	308,065 (27%)	146,035 (14%)	184,683 (13%)	132,334 (13%)	153,016 (12%)
NRCMSI	146,093 (15%)	151,452 (13%)	320,033 (31%)	435,288 (32%)	348,689 (34%)	424,275 (33%)
Poverty Relief	11,689 (1.2%)	13,812 (1.2%)	7,526 (0.7%)	11,113 (0.8%)	5,723 (0.6%)	7,390 (0.6%)
Commercial medical insurance	727 (<0.1%)	843 (<0.1%)	841 (<0.1%)	1,088 (<0.1%)	1,020 (0.1%)	1,180 (<0.1%)
Publicly funded medical care	2,087 (0.2%)	2,195 (0.2%)	2,285 (0.2%)	22,590 (1.6%)	3,195 (0.3%)	21,996 (1.7%)
No medical insurance	142,025 (15%)	187,350 (16%)	189,083 (19%)	259,947 (19%)	154,032 (15%)	194,626 (15%)
Other Social medical insurance	7,222 (0.8%)	7,531 (0.7%)	11,207 (1.1%)	12,939 (0.9%)	8,978 (0.9%)	10,138 (0.8%)
Other insurance	83,083 (8.8%)	101,898 (8.9%)	37,679 (3.7%)	42,533 (3.1%)	66,237 (6.5%)	107,841 (8.3%)

Note: Median (IQR) for numeric variables; n (%) for character variables; Clean dataset means raw dataset after cleaning missing and outlier values; CNY: Chinese Yuan, UEBMI: Urban Employment-based Basic Medical Insurance, URBMI: Urban Resident-based Basic Medical Insurance, NRCMSI: New Rural Cooperative Medical Scheme Insurance.

Table S2. Regression results of difference-in-difference event study based on data of 2019 and 2020

Week to reopen	Log(Total Expenses)				Log(Out of Pocket)				Log(Claiming Expenses)			
	Coef	S.E.	95% Conf. Int.		Coef	S.E.	95% Conf. Int.		Coef	S.E.	95% Conf. Int.	
			Lower	Upper			Lower	Upper			Lower	Upper
ref:-1	0				0				0			
-3	0.031	0.0216	-0.011	0.074	0.326	0.3073	-0.276	0.928	0.141	0.2254	-0.301	0.583
-2	0.016	0.0278	-0.038	0.071	0.366	0.3049	-0.231	0.964	0.123	0.2273	-0.322	0.569
0	0.167 ***	0.0459	0.077	0.257	0.363	0.3927	-0.407	1.133	0.529	0.3224	-0.103	1.161
1	0.141 ***	0.0425	0.058	0.225	0.321	0.4195	-0.501	1.144	0.447	0.3225	-0.185	1.080
2	0.137 ***	0.0395	0.060	0.214	0.327	0.3763	-0.410	1.065	0.337	0.2961	-0.243	0.917
3	0.149 ***	0.0436	0.063	0.234	0.340	0.3693	-0.384	1.064	0.221	0.2872	-0.342	0.784
4	0.139 ***	0.0487	0.044	0.235	0.395	0.3516	-0.294	1.084	0.253	0.2167	-0.172	0.678
5	0.142 ***	0.0442	0.055	0.228	0.397	0.3500	-0.289	1.083	0.296	0.1960	-0.089	0.680
6	0.136 ***	0.0421	0.053	0.218	0.317	0.3302	-0.330	0.964	0.375	0.2096	-0.036	0.786
7	0.130 ***	0.0430	0.045	0.214	0.360	0.3497	-0.325	1.046	0.384	0.2014	-0.011	0.778
8	0.130 ***	0.0440	0.043	0.216	0.269	0.3787	-0.474	1.011	0.512	0.3018	-0.080	1.103
9	0.122 ***	0.0385	0.046	0.197	0.355	0.4101	-0.449	1.159	0.588	0.3605	-0.118	1.295
10	0.095 ***	0.0263	0.043	0.146	0.381	0.4843	-0.568	1.330	0.706	0.4686	-0.212	1.625
11	0.134 ***	0.0364	0.063	0.206	0.485	0.4491	-0.396	1.365	0.809	0.4612	-0.095	1.713
12	0.126 ***	0.0379	0.052	0.201	0.424	0.4378	-0.434	1.283	0.926	0.4977	-0.050	1.901
13	0.123 ***	0.0349	0.054	0.191	0.344	0.4150	-0.469	1.157	0.953	0.4941	-0.015	1.922
14	0.118 ***	0.0390	0.041	0.194	0.184	0.3444	-0.491	0.859	0.864 **	0.4242	0.033	1.696
15	0.109 ***	0.0337	0.043	0.175	0.114	0.3499	-0.572	0.800	0.797	0.4243	-0.034	1.629
16	0.087 **	0.0360	0.017	0.158	0.103	0.3870	-0.655	0.861	0.737	0.3995	-0.046	1.520
17	0.103 ***	0.0369	0.030	0.175	0.120	0.3646	-0.595	0.834	0.726	0.3732	-0.006	1.457
18	0.122 ***	0.0361	0.051	0.193	0.113	0.3643	-0.601	0.827	0.797	0.4057	0.002	1.593
19	0.125 ***	0.0341	0.058	0.192	0.114	0.3518	-0.575	0.804	0.790	0.3938	0.018	1.562
20	0.117 ***	0.0389	0.041	0.193	0.081	0.2952	-0.497	0.660	0.768 **	0.3552	0.072	1.464
21	0.117 ***	0.0350	0.049	0.186	-0.012	0.2391	-0.481	0.457	0.800 **	0.3712	0.072	1.528
22	0.140 ***	0.0406	0.060	0.219	-0.008	0.2052	-0.410	0.394	0.852 **	0.3611	0.144	1.559

Week to reopen	Log(Total Expenses)				Log(Out of Pocket)				Log(Claiming Expenses)			
	Coef	S.E.	95% Conf. Int.		Coef	S.E.	95% Conf. Int.		Coef	S.E.	95% Conf. Int.	
			Lower	Upper			Lower	Upper			Lower	Upper
23	0.103 **	0.0404	0.024	0.183	0.020	0.1548	-0.283	0.324	0.794 **	0.3816	0.046	1.542
24	0.108 **	0.0430	0.024	0.193	0.017	0.1436	-0.265	0.298	0.810 **	0.3707	0.083	1.536
25	0.095 **	0.0410	0.014	0.175	0.014	0.1424	-0.265	0.294	0.794 **	0.3559	0.096	1.491
26	0.074	0.0480	-0.020	0.168	-0.022	0.1713	-0.358	0.314	0.859 **	0.3782	0.118	1.600
27	0.058	0.0335	-0.008	0.124	-0.073	0.2069	-0.478	0.333	0.966	0.4917	0.002	1.930
28	0.060	0.0310	-0.001	0.121	-0.103	0.2217	-0.538	0.331	1.041	0.5541	-0.045	2.127
29	0.031	0.0400	-0.047	0.110	-0.096	0.2302	-0.547	0.355	1.013	0.5345	-0.035	2.061
30	0.030	0.0371	-0.043	0.103	-0.156	0.2349	-0.616	0.305	1.067	0.5282	0.031	2.102
31	0.036	0.0337	-0.030	0.102	-0.200	0.2442	-0.678	0.279	1.120 **	0.5300	0.082	2.159
32	0.043	0.0305	-0.016	0.103	-0.455	0.4758	-1.388	0.477	1.114	0.5903	-0.043	2.271
33	0.060	0.0297	0.002	0.118	-0.278	0.3408	-0.946	0.390	1.196 **	0.5550	0.108	2.284
34	0.059	0.0318	-0.003	0.122	-0.254	0.3110	-0.864	0.355	1.063 **	0.4938	0.095	2.030
35	0.056	0.0295	-0.002	0.113	-0.195	0.2912	-0.766	0.376	0.830 **	0.4067	0.033	1.628
36	0.033	0.0257	-0.017	0.084	-0.131	0.2330	-0.588	0.325	0.465	0.2645	-0.054	0.983
37	0.030	0.0270	-0.022	0.083	-0.075	0.2194	-0.505	0.355	0.339	0.2412	-0.133	0.812
38	0.039	0.0297	-0.019	0.097	-0.084	0.1932	-0.463	0.295	0.300	0.1690	-0.032	0.631
39	0.042	0.0286	-0.014	0.098	-0.004	0.1615	-0.321	0.312	0.159	0.0875	-0.013	0.330
40	0.028	0.0271	-0.025	0.082	-0.044	0.1581	-0.354	0.265	0.193	0.0999	-0.002	0.389
Fixed-effects												
Week	Yes				Yes				Yes			
Hospitals	Yes				Yes				Yes			

Notes: The robust standard error (S.E.) from clustering to hospital is shown. *, ** and *** denote the significance at the level of 10%, 5% and 1%, respectively.

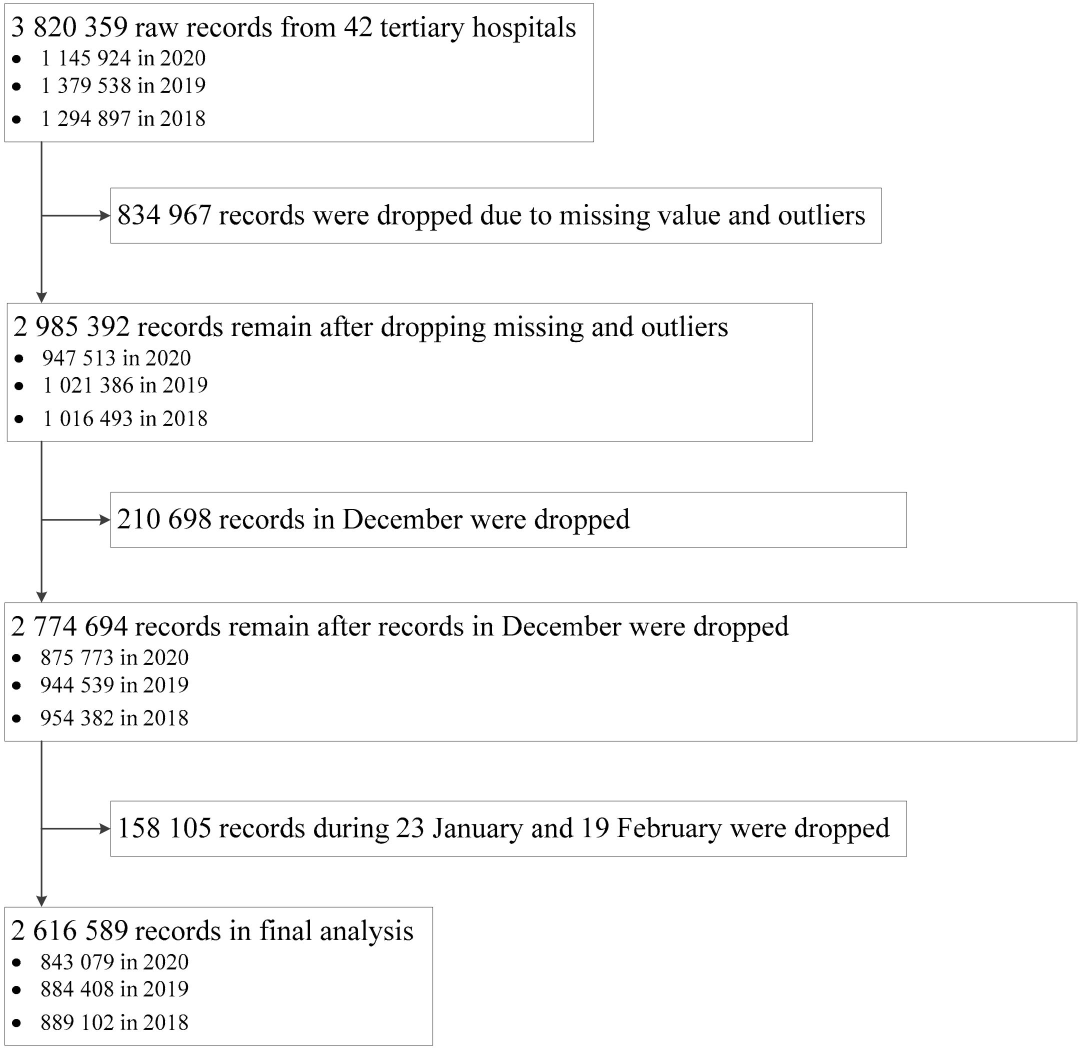

Figure S1. Flowchart of the study sample

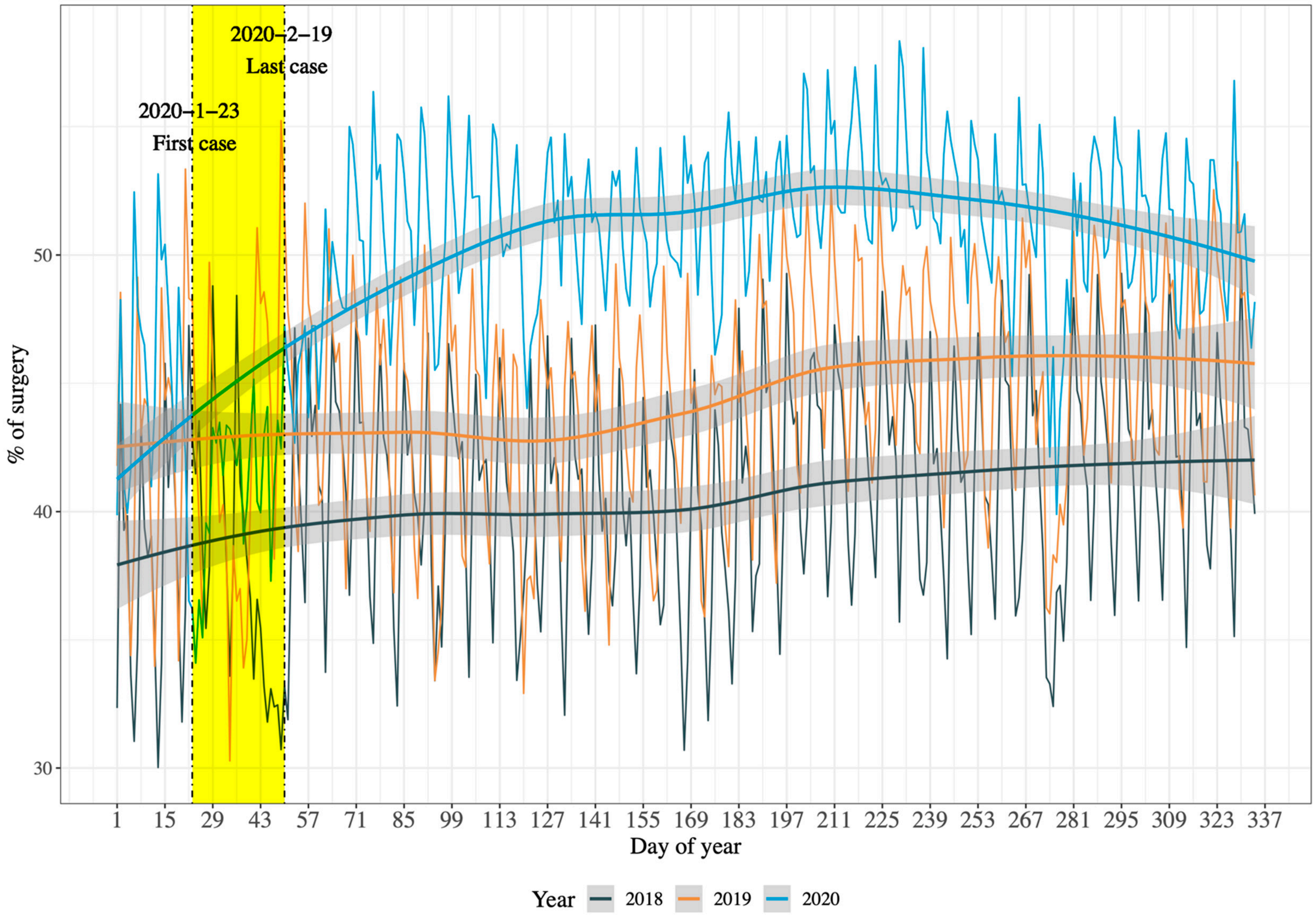

Figure S2 Percentage of patients surgery among 2018, 2019 and 2020, the solid curves are the smoothed curves fitted with the locally weighted regression, and the gray shaded area is the confidence interval of the fitted curve.

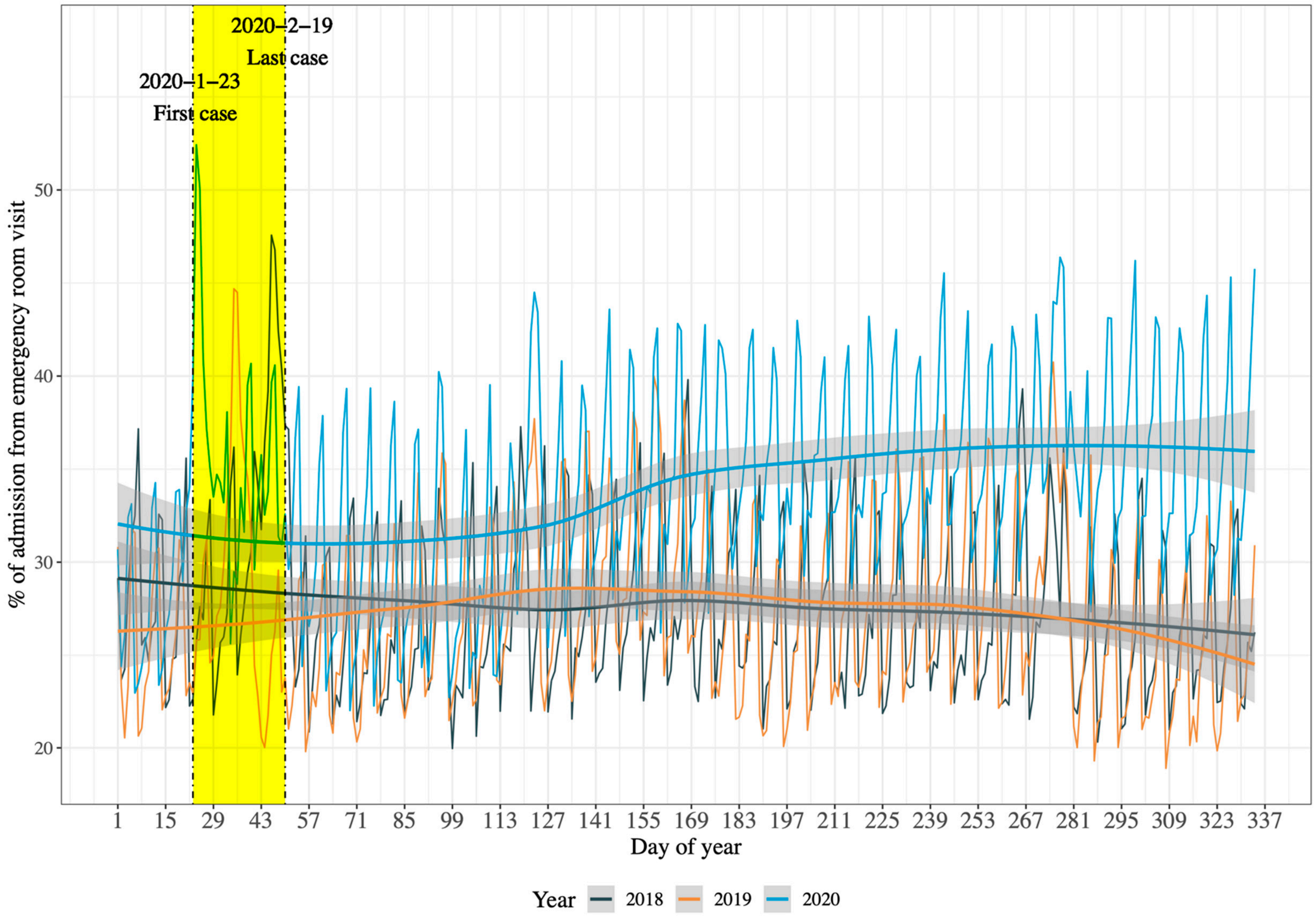

Figure S3. Percentage of admission from emergency room visit among 2018, 2019 and 2020, the solid curves are the smoothed curves fitted with the locally weighted regression, and the gray shaded area is the confidence interval of the fitted curve.
